# A Comprehensive Coordinator Supported Hepatitis C Virus Testing and Linkage to Treatment Program at Kaiser Permanente Mid-Atlantic States

**DOI:** 10.3390/v13112140

**Published:** 2021-10-23

**Authors:** Mary Cabell Jonas, Kevin Rubenstein, Eric Watson, Sundeep Basra, Michael Horberg

**Affiliations:** 1 Mid-Atlantic Permanente Research Institute, 2101 E. Jefferson Street, Rockville, MD 20852, USA; Kevin.B.Rubenstein@kp.org (K.R.); eric.s.watson@kp.org (E.W.); sundeep.s.basra@kp.org (S.B.); michael.horberg@kp.org (M.H.); 2 Mid-Atlantic Permanente Medical Group, Kaiser Permanente Mid-Atlantic States, 2101 E. Jefferson Street, Rockville, MD 20852, USA

**Keywords:** HCV, screening, usual-care testing, non-standardized testing, KPMAS, HCV treatment, HCV pathway

## Abstract

Since 2020, the US Preventive Services Taskforce has recommended expanding hepatitis C virus (HCV) screening to include ages 18−79, in addition to baby boomers (born 1945−1965) and those at-risk for hepatitis C virus. This retrospective cohort analysis compared patients (18 years and above) tested for HCV through usual care versus a coordinator-supported program (HCV pathway) during 2015−2018 within Kaiser Permanente Mid-Atlantic States (KPMAS). In total, 131,176 patients were tested through the HCV pathway and 128,311 through usual care (non-standardized testing). Of those tested, 1.6% (HCV pathway) and 0.5% (usual care) had chronic HCV. Of those with chronic HCV, more patients tested within the HCV pathway completed hepatic transient elastography (82.6% HCV pathway vs. 45.6% usual care; *p* < 0.001) and a gastroenterology visit (72.2% HCV pathway vs. 46.5% usual care; *p* < 0.001), and had filled prescriptions for treatment (56.5% HCV pathway vs. 40.3% usual care; *p* < 0.001). The median time to complete each step was shorter for those tested through the HCV pathway (hepatic transient elastography (26 vs. 118 days), gastroenterology visit (63 vs. 131 days), and prescription fill (222 vs. 326 days)). More patients tested through a coordinator-supported, standardized testing pathway completed the necessary testing steps, in less time, compared to usual care. These findings may inform institutions seeking to create effective population-wide testing programs for HCV and other conditions.

## 1. Introduction

The Centers for Disease Control and Prevention (CDC) estimates that over 2.4 million individuals in the US are infected with hepatitis C virus (HCV), with cases across all ages [1,2,3,4].Estimates suggest that half of all individuals infected with HCV are unaware of their infection [5]. In 2014, the US Preventive Services Task Force (USPSTF) recommended that all baby boomer-age (born 1945−1965) patients and those at-risk receive one-time HCV testing [6]. In March 2020, a new recommendation to test all adults aged 18–79 and pregnant women was released [7]. As such, in a fairly short time period, healthcare providers were tasked with testing one of the largest patient groups (baby boomers), a growing at-risk population, and nearly every adult patient (those aged 18−79) [2,8]. Providers have deployed a variety of strategies during the past 6 years to improve testing rates; these include reflex testing (HCV Antibody (Ab) to HCV RNA), testing across clinical sites (ambulatory, inpatient, ED, and urgent care), electronic medical record alerts and order sets, and communication campaigns [9,10,11]. Modeling studies in the literature indicate that HCV screening and care programs which simultaneously address multiple points along the HCV care cascade result in improved outcomes and higher value than interventions targeting single steps [12]. However, many providers are still challenged with linking various HCV screening initiatives into a cohesive program that closes the gaps in screening and care [13,14].

In 2014, Kaiser Permanente Mid-Atlantic States (KPMAS) implemented a multi-step, coordinator-supported HCV cascade of testing and care (HCV pathway) to close care gaps and screen the baby boomer-age population; prior research explains this program in detail and indicates success in both closing care gaps and screening the baby boomer-age population [15,16]. However, a comprehensive analysis comparing the HCV pathway versus the usual care within the same time period had not yet been undertaken. We sought to understand whether patients tested through the HCV pathway completed milestones typical to HCV care more rapidly than in usual care, as well as to understand the impact of the HCV pathway on accessing direct-acting antiviral (DAA) treatment. These two pieces of information would clarify the comprehensive impact of the HCV pathway on the patient testing continuum, including accessing DAA treatment, and would inform other health systems on how to approach universal HCV screening.

## 2. Materials and Methods

### 2.1. Study Population

Kaiser Permanente Mid-Atlantic States (Kaiser Foundation Health Plan and the Mid-Atlantic Permanente Medical Group) is an integrated care delivery system serving over 700,000 members in Maryland, Virginia, and the District of Columbia.

To determine the impact of the KPMAS HCV pathway in reducing the risk of gaps in HCV screening, we conducted an observational study on KPMAS health plan members aged 18+ enrolled at any time during the study period of 1 January 2015–31 December 2018, with an HCV Ab test result. For the analyses of steps following the testing, we included members whose HCV Ab test and confirmatory HCV RNA test results were positive. We excluded members known to be living with HIV or newly diagnosed with HIV during the testing pathway because HCV/HIV co-infected patients are engaged in care differently.

### 2.2. HCV Pathway

The KPMAS HCV pathway has been described in detail in prior publications but is explained briefly here: the HCV pathway is initiated with a unique, trackable lab order [15,16]. The order can be placed for any patient. For baby boomer and certain at-risk patients, there are Best Practice Advisory alerts in the electronic medical record (Figure S1, Supplementary Materials) [15,16]. Briefly, patients are tested for HCV Ab and if the returned results are positive, samples are automatically reflex-tested for HCV RNA, hepatitis B surface antigen, and HIV. Patients found to be positive for the HIV antibody are triaged to infectious disease for care and do not proceed along the HCV pathway (the KPMAS infectious disease care team will complete the pathway after HIV viral suppression is achieved; for KPMAS, 92% achieve HIV viral suppression through antiretroviral therapy and 90% of HIV/HCV co-infected patients have been treated for HCV) [17,18] [unpublished data]. A research nurse coordinator reaches out via phone to convey the HCV diagnosis, orders follow-up tests (hepatitis A IgG, hepatitis B surface antibody, hepatitis B core antibody, hepatic function panel, prothrombin time with INR, complete blood count, creatinine, antinuclear antibody with IFA, and hepatitis C genotype), and schedules hepatic transient elastography (FibroScan; hepatic transient elastography or TE). Once tests are complete, the research nurse coordinator shares the results and schedules a gastroenterology appointment. The HCV pathway formally ends at the gastroenterology appointment. The gastroenterologist assesses the patient for treatment and works with the clinical pharmacy team to initiate a DAA regimen. The clinical pharmacy team supports the patient through their DAA regimen; descriptions of this process are beyond the scope of this manuscript. Throughout the process, the coordinator utilizes multiple outreach modes and attempts to ensure that the patient completes the care steps.

We compared the HCV pathway to usual care, which is defined as a physician ordering each test individually, conveying the results as received, selecting and ordering follow-up labs, ordering and arranging for liver assessment (hepatic transient elastography or liver biopsy), and referring patients to gastroenterology. Follow-ups and reminders are carried out by clinic staff.

### 2.3. Variables

All study data were collected from the KPMAS electronic health record, which contains information on patient demographics, laboratory orders/results, medical office visits, medical procedure orders and results, and prescription medication fills. Study subjects were categorized into the “HCV Pathway” group or the “usual care” group according to whether they first received a pathway HCV Ab lab order or a usual care HCV Ab lab order during the study period (identified by their respective lab order codes). Our outcomes of interest were hepatic transient elastography completed as measured by procedure results; gastroenterology (GI) department visit completed; and HCV treatment prescription filled. Demographics and behavior information was recorded at the time of the HCV Ab lab result, including age (categorized by age and by baby boomers born 1945–1965), sex, self-reported race, pregnancy at the time of testing, insurance type (commercial, Medicaid, Medicare, or other), intravenous (IV) drug use, and whether the member is a man who has sex with men (MSM).

### 2.4. Statistical Analysis

Descriptive statistics in the form of counts and percentages were computed for member demographics and behaviors in the two groups of patients: those whose first HCV Ab result was from an HCV pathway order and those whose first HCV Ab result was from a usual care order. Differences in the proportions of members in each category were compared between order types using Chi-squared tests. Counts and percentages of demographics and behaviors were also computed for both test-type groups and were stratified by the year of the HCV Ab order. The same set of descriptive statistics and comparisons were computed in the subgroup of members with a positive HCV RNA result.

Among subjects with a positive HCV RNA result, time-to-event models were used to compare the risk of failing to achieve each outcome between subjects who had a pathway HCV Ab result and those who had a usual care HCV Ab result. Patients were followed from the date of HCV Ab order through to the earliest date of either the occurrence of the outcome, disenrollment from the KPMAS health plan, a change in the HCV Ab order type (switching), or after 365 days (beyond 365 days, certain tests would likely be reordered). The most common scenario for a change in the HCV Ab order type is that physicians may have initially ordered the usual care HCV Ab test, found that the patient had a positive HCV Ab result, and subsequently ordered the HCV pathway order code. This may have been due to the simplicity of placing the HCV pathway order code, which included the full set of laboratory tests and procedures, rather than placing many individual orders. Since the order type switching prevented us from being able to separate the impact of the HCV pathway from the usual care, we chose to stop the follow-ups of patients on the date of the change/switch in the HCV Ab order type. Members whose first positive HCV RNA results occurred after a change in order type or disenrollment were removed from the time-to-event analyses, as they are ineligible for all of the primary outcomes of interest during our defined follow-up time.

The numbers of days to reach each outcome was estimated using Kaplan–Meier curves with 95% confidence bands. Differences in the distributions of days comparing the HCV pathway order group and usual care order group were assessed using log-rank tests. The instantaneous risks of failing to achieve each outcome were computed using Cox proportional hazards models, with robust standard errors, adjusted for sex, self-reported race (categorized into non-Hispanic White, non-Hispanic Black, and Other or unspecified), and insurance type [19]. Patients with unknown or other insurance types were removed from the proportional hazards models.

Dataset preparation and descriptive statistics were completed using SAS software version 9.4. Time-to-event analyses and figures were created using R version 4.0.2 and the survival package [20]. This project was reviewed and approved by the KPMAS Institutional Review Board.

## 3. Results

### 3.1. Demographics of Patients Tested for HCV

Significant differences were observed across the populations tested via the HCV pathway and usual care. During the study period, roughly equivalent numbers of patients were tested through the HCV pathway (131,176) and usual care (128,311) groups (Table 1). Of those tested through the HCV pathway, a higher number and percentage were of baby boomer age (75% of HCV pathway-tested population) compared to the usual care group (30.5% of usual care-tested population). Proportionally, more females were tested for HCV through both programs (HCV pathway: 54.6% female, usual care: 67.5% female). Minor differences in race/ethnicity were seen across the testing groups, specifically within the White (HCV pathway: 29.8%, usual care: 25.8%) and Black (HCV pathway: 41.3%, usual care: 45.5%) groups. More patients with Medicare insurance were tested through the HCV pathway (18.9%) compared to usual care (9.7%).

### 3.2. Demographics of Patients with Chronic HCV

Significant differences were observed in the demographics of patients with positive HCV Ab and RNA tests (chronic HCV) tested via the HCV pathway and usual care (Table 2). A higher percentage of baby boomers were identified with chronic HCV from the HCV pathway (1646; 77.6%) versus usual care (400; 64.5%). Individuals with chronic HCV identified through the HCV pathway were more often Black (63.4%) compared to usual care (54.7%). A higher proportion of Asian individuals with chronic HCV were identified through usual care (11.5%) versus the HCV pathway (5.4%).

### 3.3. Patients Completing Steps in the Testing Pathway and Accessing DAA Treatment

Of those patients with chronic HCV and therefore eligible for progression through the cascade of care towards treatment, a significantly higher percentage of HCV pathway patients completed hepatic transient elastography (82.6%) and a gastroenterology visit (72.2%) compared to usual care patients completing hepatic transient elastography (45.6%) and a gastroenterology visit (46.5%; all *p* < 0.001; Table 3). A significantly higher percentage of patients with chronic HCV tested through the HCV pathway accessed curative DAA treatment (56.5%) compared to usual care patients (40.3%).

### 3.4. Event Completion and Time-to-Event Analyses

**Figure 1:**
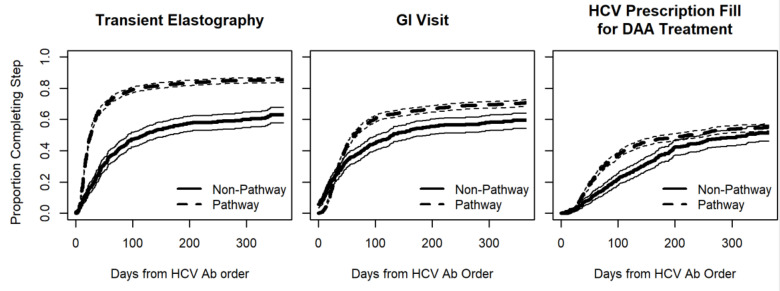
Time-to-event model for HCV care steps.

The adjusted hazard ratios of completing hepatic transient elastography, a gastroenterology visit, and DAA treatment were higher for HCV pathway patients than for usual care patients (2.43, 1.36, and 1.34 respectively; *p* < 0.001; Table 4). Unadjusted time-to-event analysis estimated that patients whose first HCV Ab order was through the HCV pathway were more likely to complete the relevant cascade of care events (hepatic transient elastography, a gastroenterology visit, and prescription fills) in a shorter time than patients whose first HCV Ab order was through usual care (Figure 1). In comparing patients tested through the HCV pathway to those tested through usual care, the estimated median time to complete hepatic transient elastography (26 vs. 118 days), a gastroenterology visit (63 vs. 131 days), and prescription fills (222 vs. 326 days) was lower for patients tested through the HCV pathway (Table S1).

## 4. Discussion

This study analyzed the overall completion and time to completion of steps along the HCV testing process, as well as the linkage to care cascade for the usual care and HCV pathway program at KPMAS. Over the four years examined, the HCV pathway was more effective than usual care in terms of the percentage of patients completing each necessary step in the testing and care cascade, and more efficient in terms of the time to complete each step. Although the HCV pathway did not formally include treatment, a higher percentage of HCV pathway patients filled DAA prescriptions. We add to the medical literature by demonstrating the longer-term success of a coordinator-driven HCV pathway in a large integrated care system and note that it can be sustained.

The KPMAS HCV pathway program was implemented in October 2014 to address the USPSTF recommendation to screen all baby boomer-age patients once for HCV [16]. A secondary goal was to establish a standardized and efficient testing process for existing HCV patients to receive the updated testing needed to initiate DAA treatment. Not surprisingly, results show that more baby boomer-age and Medicare-insured patients were tested through the HCV pathway; this was largely due to point-of-care Best Practice Advisory alerts identifying baby boomer patients and prompting the use of the HCV pathway order for testing. The operational decision to utilize the HCV pathway program to efficiently obtain up-to-date testing on existing chronic HCV patients seeking treatment, along with the higher prevalence of HCV in the baby boomer-age population, contributes to the higher percentage of patients tested through the HCV pathway having chronic HCV (1.6% HCV pathway versus 0.5% usual care). The CDC reports a prevalence of 1% for HCV in the general population [2]. One limitation of this study is that it should not be used to estimate disease prevalence within the KPMAS population. The value of this study concerns demonstrating the quality and utility of a coordinator-supported standardized testing program.

Among patients with chronic HCV, the regional consensus on the recommended course of care is to obtain, at a minimum, an assessment of liver stiffness (hepatic transient elastography) and a referral to gastroenterology for treatment assessment. Within the HCV pathway, these steps are standardized and occur in a specific order, with the support and safety net of coordinators. For usual care, the steps can occur in various sequences and some steps may not occur or may occur multiple times due to inefficiencies in the testing process (for example, multiple gastroenterology visits to review several sets of results). Results, here, show that the HCV pathway process produced a higher percentage of tested patients that completed each needed step of the recommended course of care, a result likely due to both the standardized testing pathway and the efforts of the coordinator in supporting test-ordering and patient reminders.

HCV pathway patients also complete the recommended care steps much more rapidly than in usual care. This was particularly apparent for the hepatic transient elastography procedure, in which the median time for HCV pathway patients to obtain hepatic transient elastography results was 26 days compared to the 118-day median time to results for usual care patients (Table S1). The days required for 75% of the HCV pathway population to receive hepatic transient elastography (72 days) was significantly less than the >365 days for usual care, even within an integrated care system such as KPMAS. Interestingly, for gastroenterology visits, the HCV pathway and usual care results looked similar within the first 31 days, with 25% of the visits being completed in both categories (Table S1). However, this result departs at the 50% mark, with 50% of HCV pathway patients completing the gastroenterology visit in 63 days compared to the 131 days of the usual care patients; inefficiencies in the usual care process may be the cause of these results. When physicians obtain a chronic HCV result in usual care, patients are often referred immediately to gastroenterology for next steps (data not shown). This leaves gastroenterologists to order additional needed lab testing and hepatic transient elastography, followed by additional gastroenterology follow-up visits to review all results and recommend treatment. The HCV pathway was specifically designed to complete all needed testing up-front (diagnosis and workup) to ensure the gastroenterology visit is well-spent and to eliminate unnecessary visits. These downstream efficiencies are seen in the reduced time-to-event period for HCV DAA prescription fills, in which the HCV pathway patients filled prescriptions much more rapidly than the usual care patients. Thus, although some usual care patients may access gastroenterology at the same time as a proportion of HCV pathway patients, inefficiencies in the broader usual care testing and diagnostic process delay usual care patients’ downstream initiation of DAA treatment.

Although treatment is not formally part of the coordinator-supported HCV pathway, it is an important outcome to include. Our time-to-event analyses demonstrate that more HCV pathway patients filled DAA prescriptions and accessed these DAAs more rapidly than usual care patients, as noted above. However, there is room for improvement, as only 56.5% of HCV pathway patients and 40.3% of usual care patients accessed treatment during the timeframe assessed here. Future work will continue to focus on identifying and eliminating barriers to HCV treatment.

## 5. Conclusions

This manuscript outlined a successful, scalable, and broad HCV screening and triage-to-treatment program that closes the known testing gaps for patients and enables the completion of the needed steps in less time than in usual care. The HCV pathway is effective in screening baby boomer and non-baby boomer-age adult patients, as well as efficient in triaging patients more rapidly to treatment. The program described here is suitable to support medical providers and health systems in keeping pace with the HCV USPSTF recommendations and enables organizations to advance both the HCV screening and cure goals released by the World Health Organization to move towards HCV elimination [21].

## Figures and Tables

**Table 1:** Demographics of individuals tested for HCV through the HCV pathway vs. usual care.

		HCV Pathway	Usual Care	*p*-Value
**Total 2015–2018**	**Patient**	131,176 (100.0%)	128,311 (100.0%)	
Age group	18–19	1145 (0.9%)	3987 (3.1%)	<0.001
	20–29	10,804 (8.2%)	34,088 (26.6%)	<0.001
	30–39	9862 (7.5%)	27,794 (21.7%)	<0.001
	40–49	8550 (6.5%)	19,107 (14.9%)	<0.001
	50–59	50,164 (38.2%)	22,030 (17.2%)	<0.001
	60–69	43,926 (33.5%)	16,663 (13.0%)	<0.001
	70–79	6481 (4.9%)	3907 (3.0%)	<0.001
	80–89	232 (0.2%)	693 (0.5%)	<0.001
	90–99	12 (0.0%)	41 (0.0%)	<0.001
	100+	0	1 (0.0%)	0.312
Baby boomer	Baby boomer	98,331 (75.0%)	39,134 (30.5%)	<0.001
	Not baby boomer	32,845 (25.0%)	89,177 (69.5%)	<0.001
Sex	Female	71,593 (54.6%)	86,575 (67.5%)	<0.001
	Male	59,583 (45.4%)	41,736 (32.5%)	<0.001
Pregnancy	Pregnancy during testing	403 (0.6%)	3459 (4.0%)	<0.001
Race	American Indian/Alaskan native	714 (0.5%)	764 (0.6%)	0.084
	Asian	18,813 (14.3%)	15,950 (12.4%)	<0.001
	Black/African American	54,201 (41.3%)	58,420 (45.5%)	<0.001
	Native Hawaiian/ Pacific islander	388 (0.3%)	346 (0.3%)	0.21
	Unknown or not reported	17,022 (13.0%)	18,316 (14.3%)	<0.001
	White/ Caucasian	39,111 (29.8%)	33,167 (25.8%)	<0.001
	Other	927 (0.7%)	1348 (1.1%)	<0.001
Insurance	Commercial	96,326 (73.4%)	103,012 (80.3%)	<0.001
	Medicaid	9131 (7.0%)	11,356 (8.9%)	<0.001
	Medicare	24,790 (18.9%)	12,459 (9.7%)	<0.001
	Other	64 (0.0%)	120 (0.1%)	<0.001
	Unknown	865 (0.7%)	1364 (1.1%)	<0.001
IV drug use	No or unknown	131,087 (99.9%)	128,291 (100%)	<0.001
	Yes	89 (0.1%)	20 (0.0%)	<0.001
Sexual history: men who have sex with men (MSM)	No or unknown	128,481 (97.9%)	125,040 (97.5%)	<0.001
	Yes	2695 (2.1%)	3271 (2.5%)	<0.001

**Table 2:** Demographics of individuals tested positive for chronic HCV (HCV Antibody-positive and HCV RNA-positive; monoinfected): HCV pathway vs. usual care.

		HCV Pathway	Usual Care	*p*-Value
**Total 2015–2018**	**Patients**	2122 (100.0%)	620 (100.0%)	
	18–19	3 (0.1%)	1 (0.2%)	0.909
	20–29	86 (4.1%)	33 (5.3%)	0.172
	30–39	133 (6.3%)	64 (10.3%)	0.001
	40–49	155 (7.3%)	85 (13.7%)	<0.001
	50–59	757 (35.7%)	203 (32.7%)	0.178
	60–69	892 (42.0%)	190 (30.6%)	<0.001
	70–79	86 (4.1%)	37 (6.0%)	0.043
	80–89	8 (0.4%)	7 (1.1%)	0.026
	90–99	2 (0.1%)	0 (0%)	0.444
	100+	0 (0%)	0 (0%)	
Baby boomer	Baby boomer	1646 (77.6%)	400 (64.5%)	<0.001
	Not baby boomer	476 (22.4%)	220 (35.5%)	<0.001
Sex	Female	803 (37.8%)	249 (40.2%)	0.296
	Male	1319 (62.2%)	371 (59.8%)	0.296
Pregnancy	Pregnancy during testing	11 (1.4%)	8 (3.2%)	0.042
Race	American Indian/Alaskan native	11 (0.5%)	6 (1.0%)	0.21
	Asian	114 (5.4%)	71 (11.5%)	<0.001
	Black/African American	1346 (63.4%)	339 (54.7%)	<0.001
	Native Hawaiian/ Pacific islander	3 (0.1)%	0 (0%)	0.349
	Unknown or not reported	68 (3.2%)	26 (4.2%)	0.234
	White/Caucasian	574 (27.0%)	174 (28.1%)	0.618
	Other	6 (0.3%)	4 (0.6%)	0.188
Insurance	Commercial	1251 (59.0%)	405 (65.3%)	0.004
	Medicaid	384 (18.1%)	83 (13.4%)	0.006
	Medicare	463 (21.8%)	125 (20.2%)	0.376
	Other	5 (0.2%)	1 (0.2%)	0.727
	Unknown	19 (0.9%)	6 (1.0%)	0.868
IV drug use	No or unknown	2085 (98.3%)	618 (99.7%)	0.009
	Yes	37 (1.7%)	2 (0.3%)	0.009
Sexual history: men who have sex with men (MSM)	No or unknown	2107 (99.3%)	603 (97.3%)	<0.001
	Yes	15 (0.7%)	17 (2.7%)	<0.001
DAA treatment ^1^	No treatment	924 (43.5%)	370 (59.7%)	<0.001
	treatment	1198 (56.5%)	250 (40.3%)	<0.001

^1.^ Direct-acting antiviral.

**Table 3:** Percentage of chronic HCV patients who completed each step of the testing cascade: HCV pathway vs. usual care.

	HCV Pathway	Usual Care	*p*-Value
**Total Tested**	**131,176**	**128,311**	<0.001
HCV Antibody-positive ^1^	4127 (3.1%) ^1^	2220 (1.7%) ^1^	<0.001
Chronic HCV and HIV co-infected ^2^	23 (0.6%) ^2^	0 ^2^	<0.001
Chronic HCV mono-infected (HCV Ab-positive, HCV RNA-positive) ^3^	2122 (51.4%) ^3^	620 (27.9%) ^3^	<0.001
Hepatic transient elastography ^3^	1753 (82.6%) ^3^	283 (45.6%) ^3^	<0.001
GI visit ^3^	1532 (72.2%) ^3^	288 (46.5%) ^3^	<0.001
DAA treatment ^3^	1198 (56.5%) ^3^	250 (40.3%) ^3^	<0.001

^1.^ Percentage of those tested. ^2.^ Percentage of those HCV Ab positive ^3.^ Percentage of those mono-infected with HCV.

**Table 4:** Among chronic HCV monoinfected patients, Cox proportional hazards models comparing the instantaneous risk of completing steps along the HCV care cascade between patients in the HCV pathway and patients undergoing usual care.

	Hazard Ratio ^1^	95% Lower Confidence Limit	95% Upper Confidence Limit	*p*-Value
**Hepatic transient elastography**				
HCV pathway (compared to usual care)	2.43	2.15	2.75	<0.001
**Gastroenterology visit**				
HCV pathway (compared to usual care)	1.36	1.18	1.56	<0.001
**HCV prescription fill**				
HCV pathway (compared to usual care)	1.34	1.16	1.54	<0.001

^1.^ Hazard ratio from Cox proportional hazards models adjusting for sex, self-reported race, and insurance type.

## Data Availability

The data presented in this study are available on request from the corresponding author.

## Supplementary Materials

The following are available online at https://www.mdpi.com/article/10.3390/v13112140/s1, Figure S1: The HCV pathway and Table S1: Distribution of the estimated time from the first HCV antibody order during the study period to the outcome event. Figure S1 describes the cascade of care by describing the various steps under the HCV pathway at Kaiser Permanente Mid-Atlantic States. Table S1 compares the time to completion under the usual standard of care to the HCV pathway.
