# Supplementary material for: A Comprehensive Coordinator Supported Hepatitis C Virus Testing and Linkage to Treatment Program at Kaiser Permanente Mid-Atlantic States
Source: Viruses. 2021 Oct 23;13(11):2140. doi: 10.3390/v13112140 (PMC8619706; doi:10.3390/v13112140)
Supplement: Supplementary file 1 [file viruses-13-02140-s001.zip › viruses-1414780-supplementary.pdf]

Supplementary Figures and Tables

Figure S1: The HCV Pathway

Adapted from Jonas MC, Rodriguez CV, Redd J, et al. Streamlining screening to treatment: the Hepatitis C cascade of care at Kaiser Permanente Mid-Atlantic States. Clin Infect Dis 2016;62(10):1292; with permission.

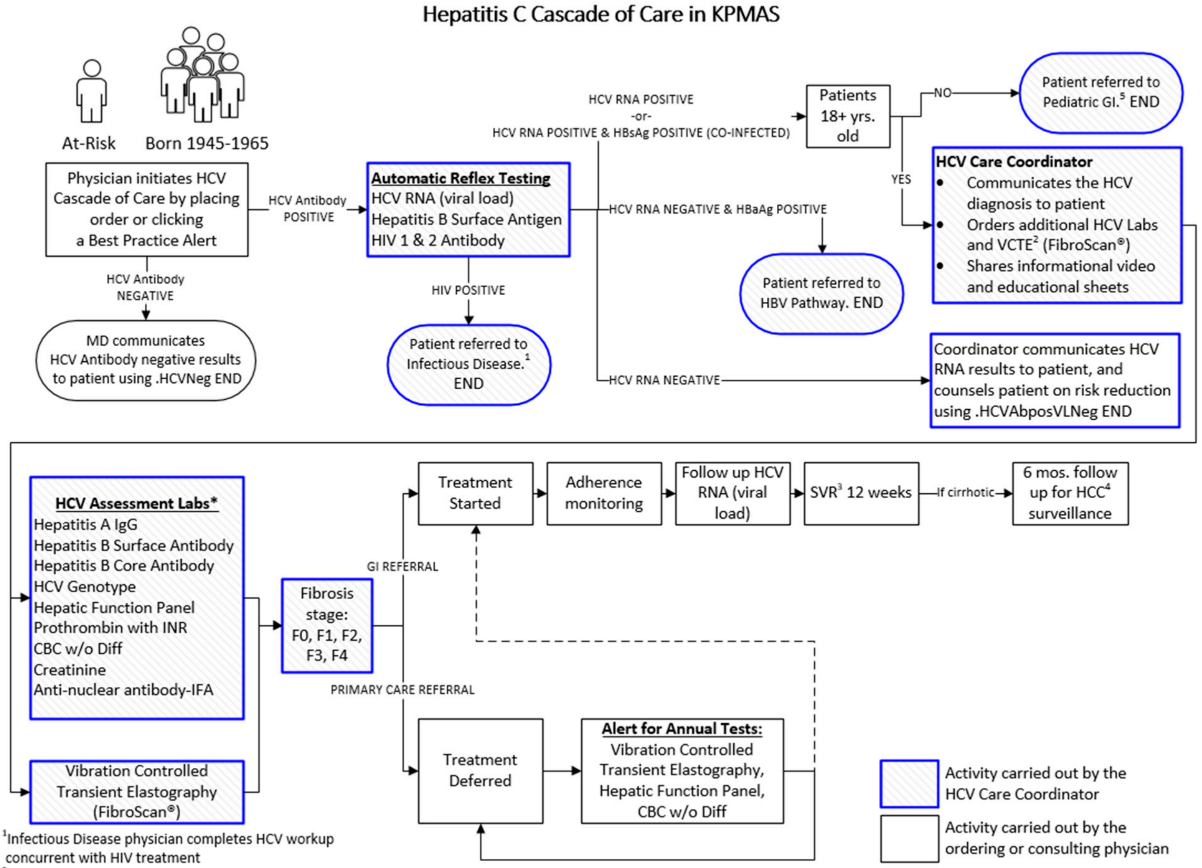

[1]Infectious Disease physician completes HCV workup concurrent with HIV treatment
[2]Vibration Controlled Transient Elastography
[3]Sustained Virologic Response
[4]Hepatocellular Carcinoma
[5]Pediatric GI conducts a different workup for HCV

**If the initial HCV Pahway order was placed by a provider other than PCP, GI or ID, the Hepatitis C Order Group Post-HCV RNA labs and Fibroscan will be ordered with a co-sign to the PCP to transfer care.*

Table S1: Distribution of estimated time from first HCV antibody order during the study period to outcome event

	Usual care first order			HCV Pathway first order			
	estimated days to 25% of events completed	estimated days to 50% of events completed	estimated days to 75% of events completed	estimated days to 25% of events completed	estimated days to 50% of events completed	estimated days to 75% of events completed	P-value *
**Fibroscan Result**	40	118	> 365	14	26	72	<0.001
**GI Visit**	31	131	> 365	31	63	> 365	<0.001
**HCV RX Fill[1]**	115	326	> 365	63	222	> 365	0.001

* Log-rank test for difference in distributions of time to event between pathway and non-pathway
[1] Direct Acting Antiviral prescription
